# Portrait of Dr. Josiah Trowbridge

**Published:** 1848-07

**Authors:** 


*Portrait of Dr. Josiah Trowbridge.*—At a recent meeting of the Buffalo Medical Association, a committee were appointed to wait on Dr. Trowbridge, of this city, and solicit him to sit for his portrait, to be preserved by the Association, of which Dr. T. was the first president. We learn that in accordance with the wishes of the Association, a portrait has been executed by Mr. Wilgus, a native of Buffalo, who is deservedly eminent as an artist. Dr. Trowbridge is, we believe, the oldest practitioner in the city, a fact, however, which does not here, as it would in older cities, imply advanced years. We do but echo an universal sentiment, in expressing the fervent hope that a long period is yet to be added to his career of honor and usefulness.

